# Genetic and Molecular Basis of Feather Diversity in Birds

**DOI:** 10.1093/gbe/evy180

**Published:** 2018-08-29

**Authors:** Chen Siang Ng, Wen-Hsiung Li

**Affiliations:** 1 Institute of Molecular and Cellular Biology & Department of Life Science, National Tsing Hua University, Hsinchu, Taiwan; 2 The iEGG and Animal Biotechnology Center, National Chung Hsing University, Taichung, Taiwan; 3 Biodiversity Research Center, Academia Sinica, Taipei, Taiwan; 4 Department of Ecology and Evolution, University of Chicago

**Keywords:** feather, skin appendage, genetics, genomics, development, evolution

## Abstract

Feather diversity is striking in many aspects. Although the development of feather has been studied for decades, genetic and genomic studies of feather diversity have begun only recently. Many questions remain to be answered by multidisciplinary approaches. In this review, we discuss three levels of feather diversity: Feather morphotypes, intraspecific variations, and interspecific variations. We summarize recent studies of feather evolution in terms of genetics, genomics, and developmental biology and provide perspectives for future research. Specifically, this review includes the following topics: 1) Diversity of feather morphotype; 2) feather diversity among different breeds of domesticated birds, including variations in pigmentation pattern, in feather length or regional identity, in feather orientation, in feather distribution, and in feather structure; and 3) diversity of feathers among avian species, including plumage color and morph differences between species and the regulatory differences in downy feather development between altricial and precocial birds. Finally, we discussed future research directions.

## Introduction

Feather first appeared in dinosaurs in the Jurassic period, around 165–150 Ma (Xu et al. 2014). One lineage of feathered theropod dinosaurs survived the mass extinction and became the ancestor of birds (Chatterjee 2015). Feather is believed to have evolved from scale, and novel scale-feather converters have just been identified (Wu et al. 2018). Compared with reptilian and avian scale, however, feather is well organized in cylindrical and tubular structures of the follicle (Prum 2005; Xu et al. 2014).

**Fig. 1.**
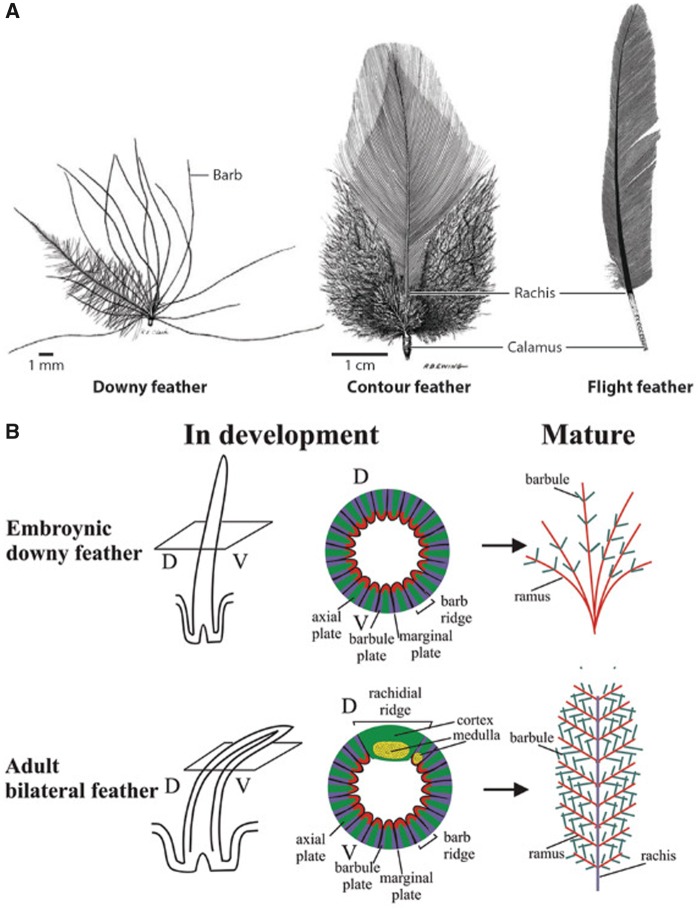
—Different types of feather in a chicken. (*A*) Downy feather, contour feather, and flight feather. (*B*) Developing and mature embryonic and adult chicken feathers. The branches in downy feathers only include the ramus and barbules, whereas most adult chicken feathers are bilaterally symmetric and include a rachis, ramus, and barbules. (*A*) Adapted from Lucas and Stettenheim (1972). (*B*) Adapted from Ng et al. (2012).

Feather diversification apparently began in theropod dinosaurs in which a wide range of feathers could already be observed (Xu et al. 2014; Chen et al. 2015). As feather is complex in structure, it has the potential to allow various phenotypic changes to evolve (fig. 1). Feather diversification allows different species of birds to be widely distributed on earth and adapt to various ecological niches in water, land, and air (Chatterjee 2015). A great diversity of feather can also be observed within a bird species, especially in domesticated birds (Bartels 2003; Chen et al. 2015; Boer et al. 2017; Domyan and Shapiro 2017). Feather, thus, provides an excellent model to study how genetic and developmental changes can evolve rapidly. It is also a rare opportunity to explore both macro- and microevolutionary questions in the same model.

Feathers play roles in heat retention, mate attraction, communication, camouflage, and skin protection (Chuong and Homberger 2003; Chuong et al. 2003; Chen et al. 2015; Persons and Currie 2015; Ruxton et al. 2017). Display or heat retention could be the original purpose of feather, but feather then adapted to flight. The evolutionary co-option of existing molecular signaling pathways through changes in their *cis*-regulatory modules allowed morphological and structural innovations of feathers to originate and evolve (True and Carroll 2002; Prum 2005; Lowe et al. 2015; Bhattacharjee et al. 2016).

Excellent reviews using feather as a model for studying evolution, regeneration, organogenesis, and signaling pathway have been published (Chuong, Chodankar, et al. 2000; Wu et al. 2004; Yu et al. 2004; Chuong et al. 2012; Lin, Wideliz, et al. 2013; Chuong et al. 2014; Chen et al. 2015; Chiu and Chuong 2015; Boer et al. 2017; Domyan and Shapiro 2017). The present review focuses on the genetic, genomic, and developmental basis underlying the diversity of feather at several levels. We first present an overview of recent studies on the transcriptomes of various feather morphotypes. Then, we summarize the genetic studies of domesticated birds—these studies not only linked phenotypes to genotypes but also attempted to identify functions of feather genes, so one can use them as candidate genes to study interspecific differences. Lastly, we discuss some recent works on interspecific comparisons.

## Diversity of Feather Morphotype

**Fig. 2.**
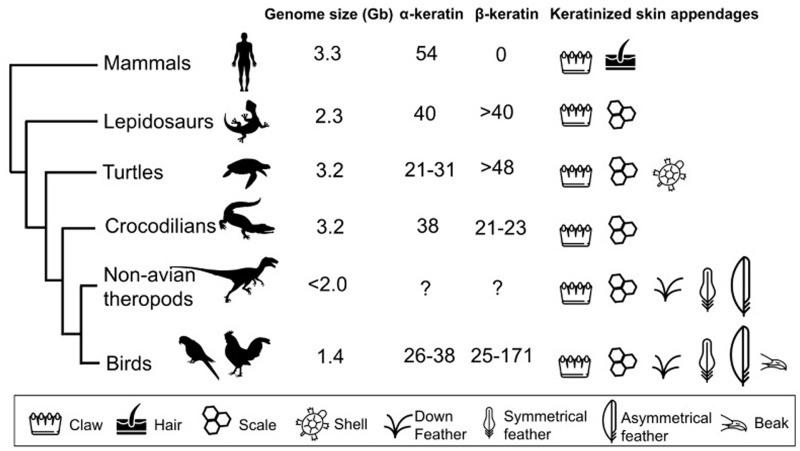
—The genome size, numbers of α- and β-keratin genes, and keratinized skin appendages of amniotes. The phylogeny of amniotes is based on molecular studies (Hedges and Poling 1999; Shen et al. 2011; Tzika et al. 2011; Chiari et al. 2012; Hedges 2012). The genome sizes of mammals, lepidosaurs, turtles, crocodilians, non-avian theropods, and birds are presented as clade-wide averages based on recent genomic and paleontological studies (Organ et al. 2007; Janes et al. 2010). The numbers of α- and β-keratin genes are based on recent genomic or developmental studies on some representative species, such as American alligator (*Alligator mississippiensis*), saltwater crocodile (*Crocodylus porosus*), green sea turtle (*Chelonia mydas*), western painted turtle (*Chrysemys picta belli*), Chinese soft-shelled turtle (*Pelodiscus sinensis*), green anole lizard (*Anolis carolinensis*), human (*Homo sapiens*), and 48 species of birds (Dalla Valle et al. 2010; Greenwold et al. 2014; Holthaus et al. 2016). Although only three major feather morphotypes are shown here, dinosaurs and birds actually have diverse morphotypes of feather, such as monofilamentous, radially branched, bilaterally branched, symmetrical flight, and asymmetrical flight feathers (Xu et al. 2014; Chen et al. 2015).

Various feather forms are found in a single bird, and seasonal change and sexual dimorphism of feather can be found in many species. The evolution of feather types is associated with the recruitment of molecular pathways (Chen et al. 2015). The presence of regulatory elements of feather development genes probably predates the origin of Dinosauria (Lowe et al. 2015). Fossil records indicate that some theropod dinosaurs already had different feather morphotypes on different body regions (fig. 2), suggesting that fairly sophisticated molecular pathways and developmental processes already existed in the ancestor of birds (Xu et al. 2014; Chen et al. 2015). Recruitments of multiple regulatory modules of scale-feather converters (SOX2, ZIC1, GREM1, SPRY2, and SOX18) may allow more complex morphogenetic events to occur (Wu et al. 2018).

Feather barbs and barbules might have been evolutionarily derived from the embryonic subperiderm, a transient layer of the skin, and co-opted with signaling and cell differentiation pathways of morphogenesis to form the branching pattern of feathers (Sawyer and Knapp 2003; Sawyer et al. 2005). This hypothesis is supported by the expression of β-keratins, EDCRP (epidermal differentiation cysteine-rich protein) and EDMTFH (epidermal differentiation protein starting with an MTF motif and rich in histidine) both in the barb and barbule cells of developing feathers and in the subperiderm layer of the embryonic epidermis, implying a topological and developmental relationship between embryonic subperiderm and feather barbs and barbules (Strasser et al. 2015; Alibardi et al. 2016). These lines of evidence suggest that cornified feather keratinocytes might have been derived from subperidermic tissues and then subsequently formed the tubular shape of the feather follicle to establish the complex branching of feathers.

The cellular and developmental mechanisms of feather formation and morphogenesis have been studied (Wu et al. 2004; Yu et al. 2004; Lin, Wideliz, et al. 2013; Chen et al. 2015). The epithelium and mesenchyme are two major components in feather follicles (Lillie and Juhn 1938; Yu et al. 2004; Yue et al. 2005). The dermal papilla and the pulp are mainly composed of mesenchymal cells (Lillie 1940; Yu et al. 2002). Branching morphogenesis is formed by invagination of the multilayered epithelium surrounding the mesenchyme in the ramogenic zone (fig. 1*B*). The rachis is formed by the fusion of barb ridges at the anterior end of the feather. Apoptosis plays an important role in feather formation. The marginal plate in the basal layer flanking each barb ridge and axial cells undergo apoptosis after the barbule plates are keratinized. The opening of feather branches is allowed by apoptosis of feather sheath and pulp epithelium in the more mature distal end (Yu et al. 2004).

Several proteins have been proposed to be involved in feather formation (Chuong et al. 2014; Chen et al. 2015). BMP, NOG, SPRY, and FGF regulate a periodic invagination that forms barb and rachis. GDF10 and GREM1 modulate the BMP signaling to regulate the periodic-branching program to control topologies of rachis and barb generative zone (Li et al. 2017). NCAM, SHH, and caspase regulate the differential cell death that forms the basal branch pattern. Moreover, WNT3A and SPRY modulate the basal branching circuit that forms radial, bilaterally symmetric, and asymmetric branching patterns. NOTCH and FGF signaling regulates differential cell adhesion and contraction of basal filopodia to form periodic branching of feather (Cheng et al. 2018). Diverse integuments and their appendages in birds are generated by regional specificities. *HOX* expression patterns show regional differences in chicken skin, suggesting that differential expression of *HOX* genes in the bird skin may determine the phenotypes of skin appendages (Chuong and Homberger 2003). Mutational changes in *HOX* genes have been shown to be associated with some well-known traits in chickens (Wang et al. 2012). This topic will be discussed in the next section.

Remodeling the expression of conserved genes is proposed to be the major source of the evolution of morphological variation (Carroll 2008; Lowe et al. 2015). Systems biology provides a new approach that can efficiently reveal gene expression patterns associated with differences in morphological developments. Transcriptomic analysis has been widely applied (Wang et al. 2009; Costa et al. 2010; Oshlack et al. 2010; Kassahn et al. 2011; Ozsolak and Milos 2011; Mutz et al. 2013). Candidate genes involved in morphogenesis, growth control, or differentiation of specific structures have been found in transcriptomic analyses of different feather types of adults and integuments of embryos in chicken and ducks (Ng et al. 2015; Li et al. 2017; Gong et al. 2018; Yang et al. 2018). In addition, bioinformatics analyses of identified genes that are associated with feather and scale differences (Chang et al. 2015; Wu et al. 2018) and miRNAs that target the cell signaling, cell adhesion, and cell structure genes required in feather morphogenesis (Zhang et al. 2013; Bao et al. 2016).

Feathers and other avian integumentary appendages such as beaks, scales, and claws are mainly composed of α- and β-keratins (Shames and Sawyer 1987; Alibardi 2016). α-Keratins are found in all vertebrates, whereas β-keratins only exist in birds and reptiles (fig. 2). Structurally, α-keratins have classical intermediate filament structures with α-helical coiled-coil structures, while β-keratins mainly have twisted β-sheet structures. Type I (acidic) and type II (basic/neutral) α-keratins form obligatory heterodimers and make 10-nm intermediate filaments (Coulombe and Omary 2002), which can be found in scutate scales, claws, beaks, and lingual nails of birds (Carver and Sawyer 1988, 1989; Shames et al. 1989; Knapp et al. 1993; Rice et al. 2013; Greenwold et al. 2014; Skieresz-Szewczyk et al. 2017), whereas β-keratins polymerize and form 3-nm β-filaments and become part of the inter-filamentous matrix in avian scales, claws, beaks, and feathers (Fraser and Parry 2008, 2011a, 2011b, 2014). Evolutionarily, α- and β-keratins are completely unrelated, and it has been proposed to rename β-keratins to corneous beta-proteins (Calvaresi et al. 2016; Holthaus et al. 2016).

**Fig. 3.**
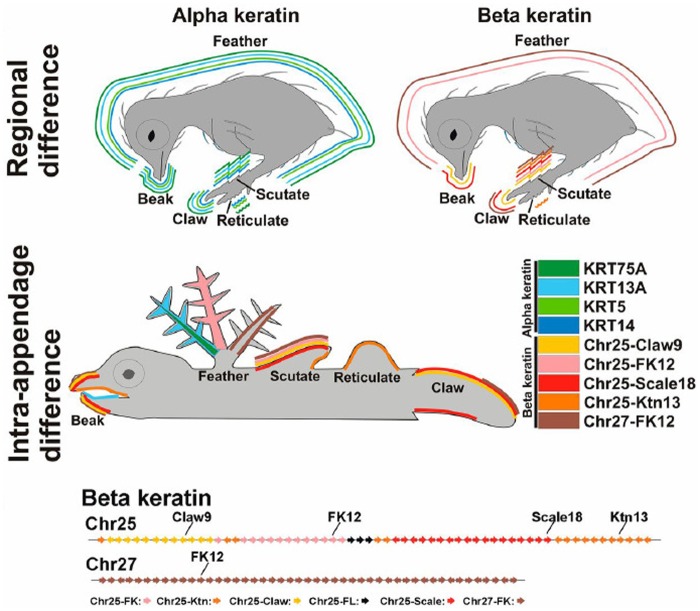
—Schematic topographic representation of differentially expressed α- and β-keratin genes in skin appendages of embryo and adult chicken based on *in situ* hybridization and RNA-seq data. Colors represent particular α- and β-keratin genes in certain appendages. Upper panel: Regional differences among skin appendages. Middle panel: Intra-appendage differences in α- and β-keratin expression. Bottom panel: Chromosomal arrangements of β-keratin genes on Chr25 and Chr27. Claw, claw-β-keratin; FK, feather-β-keratin; FL, feather-like-β-keratin; Ktn, keratinocyte-β-keratin; Scale, scale-β-keratin. Adapted from Wu et al. (2015).

Genome scanning of α- and β-keratin genes in avian genomes made the transcriptomic and evolutionary analyses possible (Greenwold and Sawyer 2010; Greenwold et al. 2014; Ng et al. 2014). Greenwold et al. (2014) conducted a microarray study on embryonic expression of α- and β-keratins in chickens at embryonic day 17 and day 19. More recent studies applied RNA-seq and in situ hybridization to map expression profiles of α- and β-keratin genes in keratinized skin appendages at embryonic day 14 and day 16 and in adult regenerating feathers in chickens (Ng et al. 2014; Wu et al. 2015) (fig. 3). Not all of these results are comparable because of different techniques used and developmental stages studied, but the data suggest that the morphological and structural diversity of avian skin appendages is contributed by combinations of α- and β-keratin genes, with intrafeather architecture complexity largely made by differential expression of feather-β-keratins, although other subfamilies of β-keratin genes are also expressed (Greenwold et al. 2014; Ng et al. 2014; Wu et al. 2015). Regulatory divergences among feather-β-keratin genes are also associated with structural differences among various portions or morphotypes of feathers (Bhattacharjee et al. 2016).

To examine functional interactions between α- and β-keratins, a retrovirus transgenic system, called RCAS, was used to ectopically express mutated α-keratin or antisense β-keratin genes in regenerating adult feathers and growing natal down (Ng et al. 2014; Wu et al. 2015). Chicken α-keratin mutants constructed based on mutations found in the human interfilament database showed abnormal phenotypes, suggesting an importance of α-keratins in feather structure (Ng et al. 2014). Moreover, interactions between α- and β-keratins were found crucial for feather development, because mutations in either type of these corneous proteins disrupted keratin networks, so that proper feather branches failed to form (Wu et al. 2015).

## Feather Diversity among Breeds of Domesticated Birds

A large repertoire of mutant phenotypes of domesticated birds has been accumulated in the past hundreds of years. In particular, chickens and pigeons display a great degree of variation in feather color, distribution and texture, body shape and size, leg length and width, and a host of other traits (Bartels 2003; Chen et al. 2015; Domyan and Shapiro 2016). As the genomes of more pigeon and chicken breeds are sequenced, candidate genes for these traits will begin to emerge, improving our understanding of the genetic basis of phenotypic variation. The availability of genome sequences, a genetic linkage map with reliable markers, functional tools, and various morphological and behavioral variations also makes the domesticated birds ideal for addressing important questions in evolutionary biology, concerning the number, location, and effect of genes underlying variation of phenotypic and adaptive traits. The following examples show the known genetic and developmental basis of feather variations in domesticated chickens or pigeons (table 1). Although many domestication traits are caused by null coding mutations (Stern and Orgogozo 2009), the majority of feather phenotypic changes occurred through *cis*-regulatory changes in domesticated birds (table 1).

**Table 1:** The Genetics of Feather Morphological Variants

Feather Variant	Bird	Possible Mutation Type	Dominance	Candidate Genes	References
Variations in feather length or regional specificity					
Muffs and beards	Chicken	*Cis-*regulatory	Incomplete dominance	*HOXB8*	Guo et al. (2016)
Crest	Chicken	*Cis-*regulatory	Incomplete dominance	*HOXC8*	Wang et al. (2012)
Variations in feather orientation					
Crest	Pigeon	Missense	Recessive	*EPHB2*	Shapiro et al. (2013)
Variations in feather distribution					
Naked neck	Chicken	*Cis-*regulatory	Incomplete dominance	*BMP12*	Mou et al. (2011)
Scaleless	Chicken	Nonsense	Recessive	*FGF20*	Wells et al. (2012)
Ptilopody	Chicken/pigeon	*Cis-*regulatory	Incomplete dominance	*TBX5*	Domyan et al. (2016)
	Pigeon	*Cis-*regulatory		*PITX1*	
Variations in feather structure					
Frizzle	Chicken	Nonframeshift deletion	Incomplete dominance	*KRT75*	Ng et al. (2012)
Silkieness	Chicken	*Cis-*regulatory	Recessive	*PDSS2*	Feng et al. (2014)

### Genetic Variations in Coloration Patterns

Feathers show distinct and colorful pigmentation patterns (Roulin 2004). Seasonal changes and sexual dimorphism of color patterns can be clearly seen at different stages of the life history of a bird. The combinations of presence, distribution, and differentiation of melanocytes can generate various color patterns. Precursors of melanocytes flow from a horizontal ring at the proximal base of the feather follicle and migrate to the barb ridges and produce pigments during feather growth (Lin, Foley, et al. 2013). Distinct genetic mechanisms cause apigmentation in different bird species. In chicken breeds, a mutation in any gene in the pigment synthesis pathway can disrupt pigment formation. Mutations that result in white plumage color of chickens include *Dominant white* (*I*), *Dun* (*I^D^*), *recessive white* (*c*), *red-eyed white* (*c^re^*), *recessive albino* (*c^a^*), *white* (*mo^w^*), *mottled* (*mo*) and *imperfect albino* (*s^al^*) (Smyth 1990). The *Dominant white* locus is known to be associated with insertion/deletion polymorphisms in the *PMEL17* gene, which encodes a melanocyte-specific protein (Kerje et al. 2004). Furthermore, an avian retroviral sequence insertion in the tyrosinase gene (*TYR*), which encodes a key enzyme required for melanin synthesis, was identified as the causative mutation of *recessive white* phenotype in chickens (Chang et al. 2006; Sato et al. 2007). Moreover, nonsynonymous substitutions C244F and R332H in the endothelin receptor B2 gene (*EDNRB2*) are responsible for the tyrosinase-independent recessive white (*mo^w^*) and mottled (*mo*) plumage phenotypes, respectively, in chickens (Kinoshita et al. 2014). In addition, the sex-linked imperfect albinism in chickens may be caused by the deletion of 1 bp (106delT) in exon 2 of *SLC45A2* (solute carrier family 45, member 2, protein) (Gunnarsson et al. 2007), resulting in a frameshift and a premature stop codon in *SLC45A2*, which encodes a membrane-associated transporter protein involved in vesicle sorting in the melanocytes.

Among pigmentation genes, functions and variations of the melanocortin 1 receptor (MC1R) gene are the most widely studied one in vertebrates. Many studies have revealed that *MC1R* is responsible for melanic polymorphisms in many avian species (Price and Bontrager 2001; Mundy 2005; Galvan and Solano 2016). Agouti signaling protein is also related to color polymorphism in mammals (Suzuki 2013). Black and white stripes are present in Barred Plymouth Rock chicken feathers where melanocytes are present in the black regions but absent in the white regions. The absence of melanocytes is due to premature differentiation, but not apoptosis (Lin, Foley, et al. 2013). A central white region with black edges in Silver Laced Wyandotte chicken feathers is not due to the presence or absence of melanocytes—it is because the expression of agouti signaling protein in the mesenchyme of white regions inhibits the maturation of melanocytes (Lin, Foley, et al. 2013).

Genetic analyses and genomic scan for coloration genes can be facilitated with the availability of reference genome sequences. Two recent studies are excellent demonstrations of this notion (Cooke et al. 2017; Vickrey et al. 2018). Rock pigeons (*Columba livia*), whose genome was sequenced and assembled (Shapiro et al. 2013; Holt et al. 2018), exhibit four color pattern phenotypes: T-check, checker, bar (ancestral), or barless. Norrie disease protein (*NDP*) gene was found to be a candidate gene for this variation (Feigin and Mallarino 2018; Vickrey et al. 2018). Genetic analyses showed that both *cis*-regulatory changes and a missense coding mutation in *NDP* are responsible for this plumage color variation. Start-codon mutations in *NDP* result in less pigmentation in barless patterned plumages. Moreover, the derived allele of *NDP* with *cis-*regulatory changes might be introgressed from the African speckled pigeon (*Columbaguinea*) into the rock pigeon possibly via hybridization events and caused allele-specific expression differences in plumages.

A causative gene of coloration was also recently mapped and characterized in budgerigars (*Melopsittacus undulatus*) (Cooke et al. 2017), which can synthesize a unique class of red, orange, and yellow polyene called psittacofulvins to make the plumage colorful and fluorescent (Hausmann et al. 2003). Budgerigars are widely used in neuroscience and behavior, and a high-coverage reference genome of budgerigar is available (Ganapathy et al. 2014). They have been extensively bred for a colorful variety of plumage phenotypes, including the wild-type green and yellow, mutant blue, and lack of psittacofulvin pigmentation and structural color white. The recessive blue color phenotypes had been known to be controlled by a Mendelian gene (Auber 1941; D'Alba et al. 2012). Genome-wide association mapping and gene-expression analysis revealed that a single amino acid substitution (R644W) at a conserved residue of an uncharacterized polyketide synthase (MuPKS), which is highly expressed in feather epithelia, is the molecular basis of the *blue* locus (Cooke et al. 2017). Interestingly, the origin of psittacofulvin synthesis is accomplished by co-opting an existing gene of polyketide synthase which likely generate conjugated fatty acids and other products originally presented in retina into plumage coloration (Mundy 2018). The genetic basis of the structural color, however, has not been studied yet.

### Variations in Feather Length or Regional Identity

Crests, muffs, and beards in chickens were thought to be variations in feather length. However, recent studies indicated that mutations in *HOX* genes could be responsible for these feather traits (Wang et al. 2012; Guo et al. 2016). Thus, changes in regional identity, not changes in feather length, might actually be the cause, because an important function of *HOX* genes is to determine the type of segment structures that form on the body segment. Ectopic expression of *HOX* genes might change the identity of the body segment, making a group of feathers grow differently.

#### Muffs and Beards

Elongated feathers grow on both sides of the face (muffs) and below the beak (beards) in muffed and bearded (Mb) chickens. It shows an autosomal incomplete dominance mode of inheritance (Serebrovsky and Petrov 1930). Genetic analyses showed that the candidate gene causing the Mb phenotype is located on chromosome 27 (Guo et al. 2016). Genome resequencing revealed a complex structural variation that may potentially lead to a higher expression level of the *HOXB8* gene in Mb chickens (Guo et al. 2016).

#### Chicken Crest

The crest in chickens is a phenotype, in which elongated feathers grow atop the head (Bartels 2003). A similar phenotype can often be observed in wild bird species, although the pigeon crest is due to a change in the growth orientation, not the length, of feathers. The chicken crest is an autosomal, incomplete dominance phenotype and is associated with cerebral hernia. Mutated genes causing chicken crest are shown to be located on the E22C19W28 linkage group (it is assigned to chromosome 33 in the galGal5 assembly) and completely associated with the *HOXC*-cluster on this linkage group (Wang et al. 2012).The chicken crest is proposed to be caused by a *cis*-acting regulatory mutation resulting in the ectopic expression of *HOXC8* (Wang et al. 2012) because the expression level of *HOXC8* is higher in the cranial skin of crested chicken embryos, but the causative mutation has not yet been identified. Understanding both Mb and crest may tell us whether ectopic expression of highly conserved HOX genes can lead to novel morphologies in vertebrate skin appendages.

### Variations in Feather Orientation

#### Pigeon Crest

The crest of pigeons is different from that of chickens. In crested pigeons, neck and occipital feathers grow with reversed polarity (Shapiro et al. 2013). A candidate gene *EPHB2*, which encodes ephrin type-B receptor 2, was identified for the pigeon crest phenotype and the derived allele is shared by a wide variety of crest breeds (Shapiro et al. 2013), suggesting that the pigeon crested breeds are all inherited from a single ancestor. The catalytic loop of the intracellular tyrosine kinase domain of mutated EPHB2 has a predicted intolerant amino acid change. How the change leads to the reversal of feather bud polarity only in neck and occipital feathers is not known.

### Variations in Feather Distribution

#### Naked Neck

The naked neck of birds completely loses feathers on the neck. An autosomal incompletely dominant allele is responsible for the trait. The naked neck (*Na*) mutation was mapped to the distal region of chicken chromosome 3 (Pitel et al. 2000)—an insertion of an intragenic region from chromosome 1 into chromosome 3 was identified as the causative mutation (Pitel et al. 2000; Mou et al. 2011). This insertion presumably carries a *cis*-regulatory element that drives the ectopic expression of *BMP12* in skin of naked neck chickens. Overexpression of *BMP12* is not enough to inhibit feather formation in body regions of the embryonic skin beside the neck area. In naked neck chickens, the developing skin of the neck becomes more sensitive to BMP signaling due to a high production level of retinoic acid (Mou et al. 2011). Many wild birds also exhibit naked neck phenotypes, but whether retinoic acid also plays an important role in generating similar phenotypes in other species is unknown.

#### Scaleless (*sc*/*sc*)

Feather and scale placodes fail to form during embryogenesis in the scaleless chicken, resulting in most feathers being absent in adult chickens (Abbott and Asmundson 1957; Dhouailly and Sawyer 1984). A single autosomal-recessive allele is responsible for the trait. The timing of reciprocal signaling between the epidermal and dermal tissues is critical for feather patterning (Sengel and Abbott 1963; Brotman 1977; Song and Sawyer 1996). Punctuated expression of *CTNNB1* and *EDAR*, which code for two proteins required for feather patterning (Widelitz et al. 2000; Drew et al. 2007), is only maintained in a very short period of time in some regions of the body of scaleless embryo (Houghton et al. 2007), diminishing pattern formation of the epidermis and causing diffusive expression of *DLL1*, the dermal condensate marker, throughout the dermis (Viallet et al. 1998). Therefore, dermal condensations cannot properly form in the skin of homozygous scaleless chickens. A stop-gain mutation in *FGF20*, a gene expressed at an early stage during feather placode development in the epidermis, was found to be the causative mutation of the scaleless phenotype (Wells et al. 2012).

#### Ptilopody

Foot feathering is observed in some wild birds as well as in domesticated pigeons and chickens. Two major-effect genes account for foot feathering in pigeons (Doncaster 1912; Wexelsen 2010). Regulatory mutations in the hindlimb-specific transcription factor gene *PITX1*, which encodes paired-like homeodomain 1, and forelimb-specific transcription factor gene *TBX5*, which encodes T-box transcription factor, have both been identified as the causative genes of ptilopody of pigeon (Domyan et al. 2016). Changes in the expression of *PITX1* and *TBX5* may partially transform hindlimb to forelimb identity and therefore form feathered feet in pigeons. Ectopically expressing *TBX5* can cause foot feathers in chickens (Domyan et al. 2016).

### Variations in Feather Structure

#### Frizzle

**Fig. 4.**
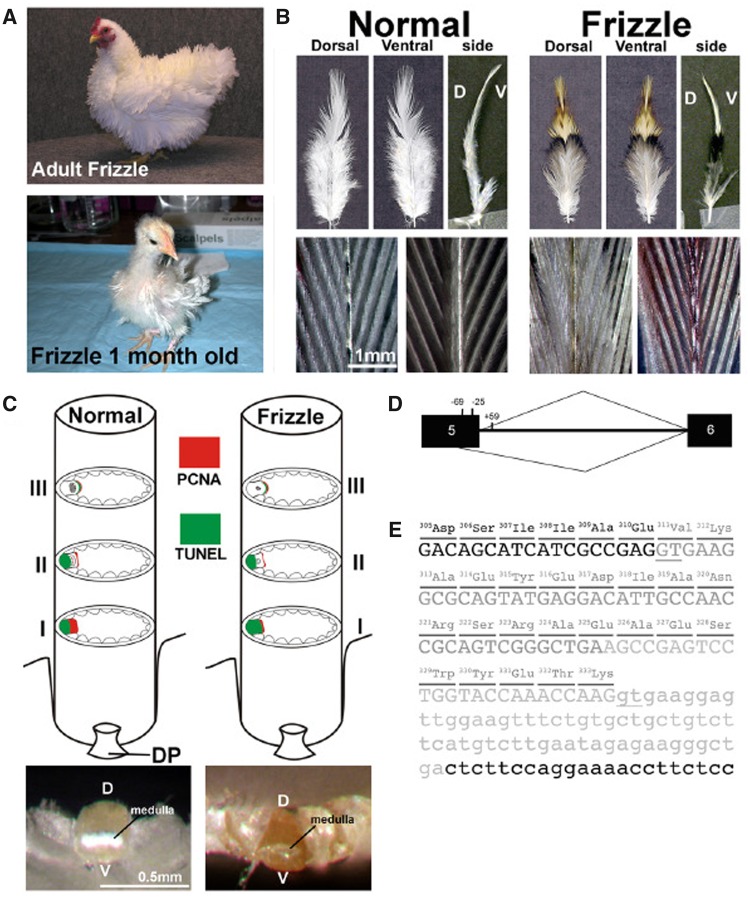
—Frizzle mutation. (*A*) Adult and 1-month-old frizzle chickens. (*B*) Comparison of body feathers of normal white leghorns and frizzle chickens in dorsal, ventral, and side views. (*C*) Upper panel: PCNA and TUNEL staining at different levels of the rachis. Lower panel: Top view of a cross section through the rachis in a pennaceous vane of body feathers. (*D*) Chicken KRT75 and the cryptic splicing site activated by the deletion that covers positions 224 of exon 5 to +59 of intron 5. Black boxes represent exon sequences; intron 5 is designated by a line. The caret designating use of the cryptic splicing site (position 269) is shown below, and the caret designating use of the authentic site is shown above the diagram of the pre-mRNA. (*E*) Partial sequence of the *F* allele of *KRT75* gene. Light gray letters show the 84-bp deletion in genomic DNA. Dark gray letters show the additional deletion in exon 5 created by a cryptic splicing site. One transcript with a 69-bp deletion is produced by the activation of the cryptic splicing site. Therefore, a protein with a deletion of 23 amino acids (positions 311–333) may be produced. Capital and small letters show parts of exon 5 and intron 5, respectively. The authentic and cryptic mRNA splicing sites are demonstrated by the underlines. Adapted from Ng et al. (2012).

Frizzle feathers have been described in some breeds of chickens (fig. 4*A*) where the contour feathers of adults all curl outward and upward (fig. 4*B*) because of defects in feather medulla formation in which the cell proliferation zone in the frizzle rachis is much narrower than that in a normal rachis (fig. 4*C*). The frizzle phenotype of chicken is an autosomal incomplete dominance trait. An analysis of single nucleotide polymorphisms on a number of pedigrees of frizzle chickens showed the causative gene in a cluster of α-keratin genes within the linkage group E22C19W28_E50C23 (it is assigned to chromosome 33 in the galGal5 assembly) (Ng et al. 2012). Sequence analysis of the gene cluster identified a 69 bp in-frame deletion in the *KRT75* gene (fig. 4*D* and *E*) (Ng et al. 2012). Overexpression of the mutated *KRT75* in normal feather follicles produced a curl rachis (Ng et al. 2012). Moreover, the hair disorder pseudofolliculitis barbae in humans is caused by mutations in KRT5, which is expressed in the keratinocytes of the companion layer, matrix, and medulla of the mammalian hair follicle (Winter et al. 2004). Overexpressing a *KRT5* mutation in regenerating contour feathers also caused distortions in feather structure. As KRT75 and KRT5 are both α-keratins, these studies revealed an important role of α-keratins in feather structure and rejected the traditional view that α-keratins are absent in feathers.

#### Silkieness

A lack of barbicel formation is responsible for the silky-feather, which is inherited as an autosomal recessive trait and is also referred to as hookless (Cole and Hollander 1939; Miller 1956). The Silkie chicken exhibits a fluffy appearance in the body contour feathers. The flight feathers of Silkie chickens are less affected, so they can form some hooklets (Jones 1921). The silky feather locus of chicken was mapped to chromosome 3 (Dorshorst et al. 2010; Feng et al. 2014). A single regulatory single nucleotide polymorphism regulating the expression of the *PDSS2* gene, which encodes decaprenyl-diphosphate synthase subunit 2, is proposed to be associated with the silkieness phenotype in the chicken (Feng et al. 2014). The silky-feather can also be found in domesticated pigeons (the Silky Fantail) but is different from the silky-feather in chickens. In the pigeon breed, the gene is named the lace-feathering locus (Cole and Hollander 1939). Hooklets are present on the barbules in the silky pigeons but are abnormally thickened.

## Diversity of Feathers among Avian Species

Evolutionary stasis (conservation) at the level of chromosomal structure and gene synteny is observed among avian genomes (Ellegren 2010). In birds, despite the presence of diverse and highly novel phenotypic features, few genomic structural changes have been identified as the cause of the phenotypic change. Moreover, no novel genes except β-keratin genes and other genes of the epidermal differentiation complex (EDC) have been found involved in feather development (fig. 2). However, regulatory innovations in feather genes have been detected (Lowe et al. 2015). Indeed, one of the avian-specific highly conserved elements might drive the novel expression pattern of *SIM1*, which encodes a transcription factor in the forelimb and may be associated with flight feather development (Seki et al. 2017).

The expansion and radiation of β-keratins are among the few significant genomic changes found to date in the avian lineage (Greenwold and Sawyer 2010; Greenwold et al. 2014; Ng et al. 2014). Sauropsid-specific β-keratins have been shown to evolve as a subclass of the EDC genes (Strasser et al. 2014). Evolutionary and comparative genomic analyses showed that avian EDCRP might have been derived from an internal highly cysteine-enriched amino acid sequence motif of EDC that existed in the common ancestor of birds and crocodiles (Holthaus et al. 2018). All subfamilies of β-keratins are located within the EDC on microchromosome 25 of chicken, and feather β-keratin genes found on other chromosomes could have originated from this cluster. Specifically, the expansion of the β-keratin gene family has been suggested to be correlated with the diversification of feathers. Feather-β-keratins are thought to have evolved from scale- or claw-β-keratins (Greenwold and Sawyer 2010). Phylogenetic and transcriptomic analyses showed that the feather-β-keratin on chromosome 7, which is phylogenetically the most basal among all feather-β-keratins (Greenwold et al. 2014) and is mainly expressed in pennaceous barbules (Kowata et al. 2014), still shares a common regulator with scale- and claw-β-keratin genes, whereas the feather-β-keratins on chromosomes 2, 6, 10, 25, and 27 have recruited chromosome-specific regulators (Bhattacharjee et al. 2016). Understanding expression patterns and functions of the different subfamilies of β-keratins is crucial to understand how gene family expansion can help organisms to adapt to their environments and lifestyles (Greenwold et al. 2014).

### Plumage Color and Morph Differences Promote Speciation

The phenotypic traits of plumage are often related to mate choice and species recognition, and divergence of plumage trait can promote speciation in birds. The genetic and molecular mechanisms of interspecific differences of plumage traits, however, are largely unknown. Plumage traits are usually very complex and involved many genes, but coloration is one of the most well-recognized and studied trait. Indeed, recent studies of two crow species in Europe have found the putative molecular pathways of plumage color trait, using genomic and transcriptomic approaches (Poelstra et al. 2014; Vijay et al. 2016).

#### Carrion Crow versus Hooded Crow

The carrion crow (*Corvus corone*) and the hooded crow (*Corvus cornix*) are two phenotypically distinct crow species in Europe. These two closely related crow species differ distinctly in their plumage colors: The plumage of carrion crows is all black, whereas that of hooded crows is gray-coated. Most of their geographic distributions do not overlap with two exceptions, one of which forms a hybrid zone, which distributes roughly from north to south through central Europe (de Knijff 2014). Because of gene flow, most genetic markers are undifferentiated between them (Haring et al. 2007; Haas et al. 2009). Some biologists classified them as two subspecies because of genome-wide genetic homogeneity and lack of complete reproductive isolation (Wolf et al. 2010), but some others considered them as full species because of apparent nonrandom mating and reduced hybrid fitness. Despite of having hybrid zones, spatial segregation of plumage coloration has remained remarkably stable over the past century. Color-assortative mating suggested that the color differentiation of plumage could have promoted speciation of these two species.

Using the high-coverage whole-genome sequences and transcriptomes of these two crow species, Poelstra et al. (2014) tried to identify the “speciation islands” from a vast majority of lowly differentiated genomic regions. One of those speciation islands is located on chromosome 18 with a size of 1.95 Mb which contains a large inversion found in carrion crows but not in hooded crows. Several genes in this genomic region that are involved in the regulation of pigmentation, visual perception, and hormonal balance were underexpressed in hooded crows, suggesting that the color and visual cue are coupled together, so that they tend to perform associative mating. In a population genomic study, Vijay et al. (2016) analyzed 124 genomes of crow populations of the *Corvus* (*corone*) spp. crow species complex. Parallelism of a sexually selected plumage phenotype can be found in several contact zones of these species (*C. corone/cornix/orientalis/pectoralis*), in which divergent selection pressures are common. The genomic regions with signatures of selection they identified are specific to different phenotypic contact zones and divergently selected candidate pigmentation genes were mostly different among populations, suggesting that selection mainly acts on the molecular pathway linked to the multigenic phenotype rather than repeatedly acts on the single gene. This phenomenon may be due to limited local genetic variations of genic targets for divergent selection to act on.

#### Color and Plumage Morphs of the White-Throated Sparrow and the Ruff

The white-throated sparrow (*Zonotrichia albicollis*) in North America shows two color morphs of their plumage: White-striped and tan-striped on their head and throat. Interestingly, birds of either sex mate mostly with individuals of the opposite sex with another color morph (Campagna 2016). Tuttle et al. (2016) applied de novo whole-genome sequencing coupled with population- and phylogenomic data and found a massive supergene responsible for both morphological and behavioral differences between the two morphs. Over 1,000 genes are highly divergent between the two morphs, many of which are candidate genes for morph-specific behavior and plumage (Tuttle et al. 2016). Phylogenomic analysis showed that the two supergene alleles originated prior to the speciation of the white-throated sparrow from its sister species Harris’ sparrow and existed in an unknown sister species. This chromosome became polymorphic in the white-throated sparrow relatively recently, probably through a past hybridization event followed by an adaptive introgression. They also found that the “white” allele carries too many deleterious mutations because of absence of recombination and genes in the inversion are underexpressed. Another supergene has also been shown to control three different forms (independents, satellites, and faeders) of body size, breeding plumage, and behavior in the ruff, *Philomachus pugnax*, a lek-breeding wading bird (Kupper et al. 2016; Lamichhaney et al. 2016). Four mutations in the satellite supergene allele that disrupt the MC1R protein may result in white tufts of satellite ruffs (Lamichhaney et al. 2016). These genomic approaches are powerful for revealing the phenotypic integrity of plumage traits and speciation of birds.

### Altricial versus Precocial Birds

Natal down, the downy feather in hatchlings, is one of the distinctive characters to discriminate between altricial and precocial birds (Starck and Ricklefs 1998). Little or no downy feathers are found in the skin of altricial hatchling, whereas downy feathers cover precocial hatchlings. The divergence of natal down development between altricial and precocial hatchlings is thought to be caused by different requirements for heat transfer and conservation (Starck and Ricklefs 1998). The naked dorsal skin of altricial hatchlings could facilitate more efficient heat transduction from their parents (Starck and Ricklefs 1998). Furthermore, saved energy of feather growth can be used for developing other organs, such as the faster brain growth of chicks (Starck and Ricklefs 1998). The phylogenetic distribution of altricial and precocial birds (Starck and Ricklefs 1998) and the discovery of a precocial avian embryo fossil indicate that the altricial phenotype evolved from the precocial phenotype.

**Fig. 5.**
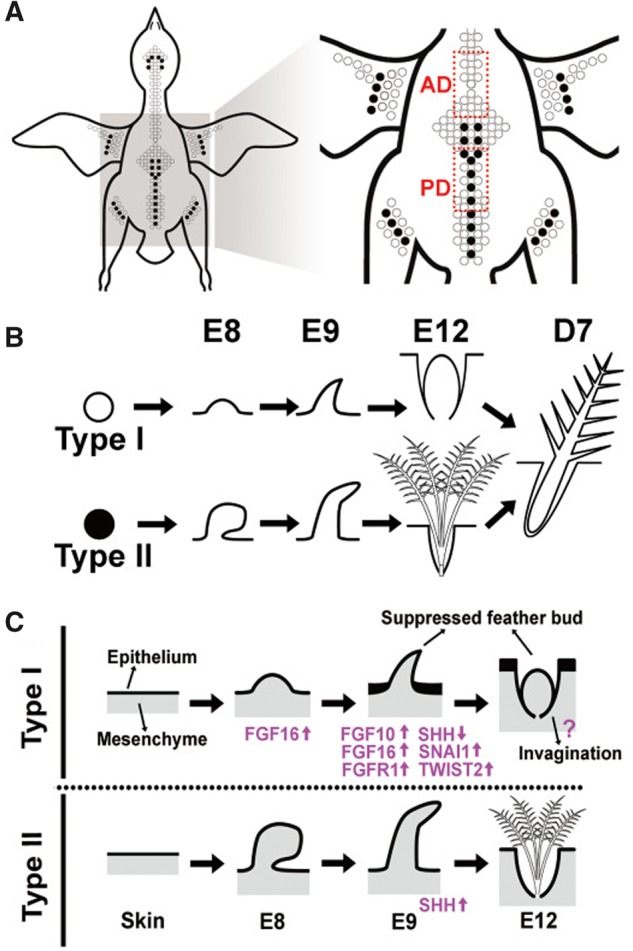
—Dorsal natal down formations in zebra finch and chicken. (*A*) Dorsal view of the feather tracts in a zebra finch hatchling. Open circles show feather buds that do not develop into downy feathers, and black circles indicate downy feather formations. (*B*) Type I (open circles) and type II (black circles) feather formations. Type I feather buds do not develop into downy feather, and contour feathers develop directly feather buds. In contrast, in the middle stripe of the posterior dorsal tract and other regions labeled with black circles, the feather buds form natal down before the growth of the contour feathers, same as the natal down formation process in chickens. (*C*) A summary diagram of types I and II feather formations, and genes involved in the down development pathway. Adapted from Chen et al. (2016).

Zebra finch (*Taeniopygia guttata*) and chicken are commonly studied altricial and precocial birds, respectively. Their genomes are sequenced and well annotated (International Chicken Genome Sequencing Consortium 2004; Warren et al. 2010). As chromosome 25 of zebra finch contains fewer feather-β-keratin genes than that of chicken, it was proposed that this could be to a consequence of the lack of natal down in Passeriformes (Greenwold and Sawyer 2010). A transcriptomic study showed that natal down of chicken mainly uses feather-β-keratin genes on chromosomes 1 and 25 (Wu et al. 2015). Chen et al. (2016) used these two birds as models for identifying the regulatory differences between altricial and precocial natal down development. In particular, in zebra finch, natal down growth is suppressed in the anterior dorsal skin but only partially suppressed in the posterior dorsal skin (fig. 5*A* and *B*), so the gene regulatory differences in natal down growth suppression and promotion can be identified by comparing the transcriptomes of the two types of skins at different developmental stages.

*SHH* (*sonic hedgehog*), the feather growth promoter (Chuong, Chodankar, et al. 2000; Chuong, Patel, et al. 2000), was found to have a higher expression level in the natal down-growing region than in naked skin (Chen et al. 2016). Moreover, both the RNA-seq and in situ hybridization data suggested that the involvement of FGF/MAPK signaling pathway suppresses *SHH* expression in naked skin. Ectopic expression of *FGF16*, the candidate suppressor, on embryonic chicken skin downregulated *SHH*, upregulated the feather growth suppressor *FGF10*, and suppressed the feather bud elongation, suggesting that the differences in natal down formation between precocial and altricial birds are due to the regulatory divergence in genes of the FGF/MAPK signaling pathway (fig. 5*C*). Moreover, a long noncoding RNA may regulate the 3′ UTR of an upstream factor of c-Myc, which is known to promote the epithelium cell proliferation in feather bud elongation (Chen et al. 2017).

According to the phylogenetic distribution, the precocial–altricial transition appears to have occurred multiple times during the evolution of birds (Starck and Ricklefs 1998; Jarvis et al. 2014; Prum et al. 2015). Birds belonging to Ciconiiformes and Gruiformes are mostly precocial but they are nested with other altricial lineages, while altricial birds of Cuculiformes and Apodiformes are clustered with other precocial lineages (Starck and Ricklefs 1998; Jarvis et al. 2014; Prum et al. 2015). It is still unknown if different mechanisms act in the natal down growth regulation in different bird lineages. It is possible that the FGF/MAPK signaling pathway was utilized as the natal down growth suppressor only in some lineages. Therefore, altricial versus precocial phenotypes may give us an outstanding opportunity to learn whether similar phenotype divergences can be caused by changes in different molecular pathways.

## A Perspective

The studies reviewed above provide a framework for future research. Many questions remain to be answered. For instance, how is the regional specificity of developing feathers generated? How are α- and β-keratin genes regulated in different types or structures of feather? How do feather follicles modulate the development of different regions to generate various feather morphotypes? Both transcriptomic and epigenomic approaches are needed to answer these questions. Studying these issues by modern sequencing technologies and bioinformatic tools can provide insights into the genomic basis underlying phenotypic diversification.

The molecular signaling in the formation of barb ridges that generate various types of feather is still largely unknown. Transcriptomic and epigenomic approaches will be needed to identify key molecular pathways and regulation. Moreover, developmental and histological techniques are also needed because feather morphogenesis is such a complex process involving precise coordination of many molecular and cellular events within the feather follicle. While genetic analysis is critical to reveal the molecular identity of many genes responsible for feather variations in domesticated birds, we should not miss their potential for controlling natural variations and should move forwards to test these genes in wild birds. Identifying molecular pathways responsible for interspecific differences has become simpler because of having more avian genomes, better molecular tools, and more accurate phylogenies. Feathers will be proven to be an excellent platform to address many biological questions at multiple levels.
